# Systematic Monitoring of Voluntary Medical Male Circumcision Scale-Up: Adoption of Efficiency Elements in Kenya, South Africa, Tanzania, and Zimbabwe

**DOI:** 10.1371/journal.pone.0082518

**Published:** 2014-05-06

**Authors:** Jane T. Bertrand, Dino Rech, Dickens Omondi Aduda, Sasha Frade, Mores Loolpapit, Michael D. Machaku, Mathews Oyango, Webster Mavhu, Alexandra Spyrelis, Linnea Perry, Margaret Farrell, Delivette Castor, Emmanuel Njeuhmeli

**Affiliations:** 1 Tulane School of Public Health and Tropical Medicine, Department of Global Health Systems and Development, New Orleans, Louisiana, United States of America; 2 Centre for HIV/AIDS Prevention Studies, Johannesburg, South Africa, Nairobi, Kenya; 3 FHI 360, Nairobi, Kenya; 4 Jhpiego, Dar es Salaam, Republic of Tanzania; 5 Zimbabwe AIDS Prevention Project, Department of Community Medicine UZ, Harare, Zimbabwe; 6 United States Agency for International Development, Washington, District of Columbia, United States of America; World Health Organization, Switzerland

## Abstract

**Background:**

SYMMACS, the Systematic Monitoring of the Voluntary Medical Male Circumcision Scale-up, tracked the implementation and adoption of six elements of surgical efficiency— use of multiple surgical beds, pre-bundled kits, task shifting, task sharing, forceps-guided surgical method, and electrocautery—as standards of surgical efficiency in Kenya, South Africa, Tanzania, and Zimbabwe.

**Methods and Findings:**

This multi-country study used two-staged sampling. The first stage sampled VMMC sites: 73 in 2011, 122 in 2012. The second stage involved sampling providers (358 in 2011, 591 in 2012) and VMMC procedures for observation (594 in 2011, 1034 in 2012). The number of VMMC sites increased significantly between 2011 and 2012; marked seasonal variation occurred in peak periods for VMMC. Countries adopted between three and five of the six elements; forceps-guided surgery was the only element adopted by all countries. Kenya and Tanzania routinely practiced task-shifting. South Africa and Zimbabwe used pre-bundled kits with disposable instruments and electrocautery. South Africa, Tanzania, and Zimbabwe routinely employed multiple surgical bays.

**Conclusions:**

SYMMACS is the first study to provide data on the implementation of VMMC programs and adoption of elements of surgical efficiency. Findings have contributed to policy change on task-shifting in Zimbabwe, a review of the monitoring system for adverse events in South Africa, an increased use of commercially bundled VMMC kits in Tanzania, and policy dialogue on improving VMMC service delivery in Kenya. This article serves as an overview for five other articles following this supplement.

## Introduction

Based on results of three clinical trials [1]–[3] the World Health Organization (WHO) and the Joint United Nations Programme on HIV/AIDS (UNAIDS) recommended scaling up voluntary medical male circumcision (VMMC) in 14 priority settings of high HIV prevalence and low levels of male circumcision [4]–[6]. In 2010, a WHO panel of experts issued “Models for Optimizing the Volume and Efficiency for Male Circumcision Services” or MC MOVE [7], which outlined “considerations” for improving efficiency while ensuring safety, depending upon relevance in the local context. For SYMMACS (the Systematic Monitoring of the Voluntary Medical Male Circumcision Scale-up in Eastern and Southern Africa), practitioners working closely with the scale-up identified six elements related to surgical efficiency in high volume settings:

1. Multiple surgical bays (using an additional bay can improve efficiency by reducing or eliminating provider time lost waiting between clients for a surgical bay to be prepared);
2. Use of kits containing pre-bundled consumables and disposable instruments (which guarantee sterility and eliminate onsite instrument sterilization);
3. Task-shifting: allowing well trained other clinical providers (such as nurses or clinical officers) to complete all steps of the VMMC procedure;
4. Task-sharing: delegating surgical tasks – scrubbing the client, administering anesthesia, and completing the suturing after mattress sutures are applied – to trained other clinical providers;
5. Use of the forceps-guided surgical method; and
6. Use of electrocautery (diathermy) to stop bleeding more quickly than suturing [7].

The objective of this study was to document the expansion of VMMC sites, the monthly fluctuations in VMMC update, and the extent of adoption of six surgical efficiency elements across the four countries. As the first of six articles in this supplement based on SYMMACS, the methodology also applies to the five articles that follow.

## Methods

### Study design

SYMMACS was a two-stage, multi-country study with two cross-sectional rounds of data collection (2011 and 2012). The two-stage sampling consisted of (1) selecting VMMC sites (fixed, outreach, or mobile) at government, NGO, faith-based, and other private facilities (except military sites) at which VMMC procedures were performed as part of comprehensive HIV prevention services in Kenya (KE), Republic of South Africa (RSA), Tanzania (TZ), and Zimbabwe (ZW); and (2) selecting both clinical providers for interview and VMMC procedures for direct observation. Each country team included 2–5 co-investigators; at least one research coordinator, one social scientist, and one clinician trained in VMMC; and a data manager.

### Sampling

To track changes in VMMC service delivery, the two-stage sampling was designed to allow for sites to be added over the observation period as they became operational. The first stage of sampling consisted of selecting VMMC sites in each country. The four countries differed in the stage of their VMMC scale-up, which affected the sampling strategy. KE (Nyanza province) had a well-established program with over 235 sites operational by December 2010. Thirty sites were randomly selected for 2011 data collection. In 2012 four of these sites were replaced because of programmatic changes and one was dropped for lack of clients, resulting in 29 sites in KE in 2012. By contrast, RSA, TZ, and ZW had only 1–3 VMMC sites by late 2010. Each country identified and selected all known or planned sites for 2011. RSA identified 10 sites, but an additional five emerged in the course of 2011. In TZ, 14 sites were identified and selected. And in ZW, 10 sites were initially identified, but four more emerged and were selected.

The 2012 sample increased in RSA, TA, and ZW to incorporate newly operational sites. In RSA, by 2012 over 80 VMMC sites were operating. SYMMACS returned to the original 15 sites, but purposively selected an additional 25 sites, for a total of 40. Criteria included selecting sites performing at least 100 VMMC per month, covering a maximum number of provinces (6 of 9), and including multiple partner organizations (9 of 14). In TZ, the team selected the same 14 sites as in 2011, but added all fixed sites known to be operational as of January 2012 (n = 10) and all outreach sites serving at least 100 VMMC clients a day (n = 5), for a total of 29 sites. (The rationale behind the minimum number of clients per day was to capture data from sites that were most active, thus capturing the experience of the greatest number of clients.) In ZW, only six of the 14 original sites were still operational in 2012. New sites were selected to include each outreach team and to cover all 10 provinces, resulting in 24 sites in 2012.

The second stage of sampling included selection of both providers and VMMC procedures for observation. The provider survey sampled all clinical staff providing VMMC services over two days of data collection per site. For VMMC procedures, the team attempted to observe up to 10 VMMC procedures per site, starting with the first operation on Day 1 and continuing with each procedure available for observation from the start. Where client load was low, fewer than 10 per site were observed.

### Data collection

**Figure 1:**
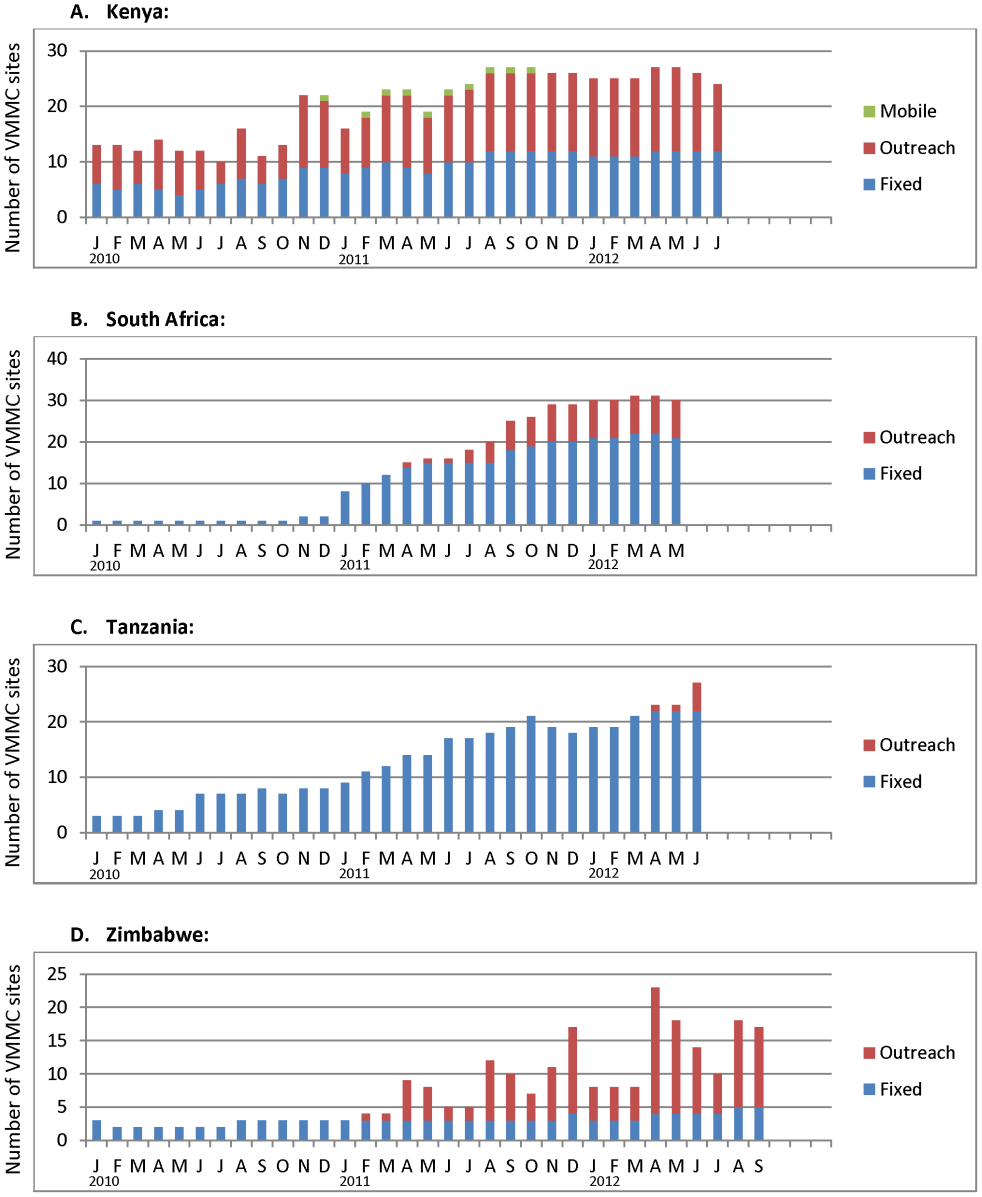
Number of operational VMMC sites by month, type of site, and country, January 2010 - mid 2012. In this and all figures following, “Kenya” refers to Nyanza Province only

Draft instruments were vetted with members of the country teams and external advisors. All country teams underwent a one-week training workshop on data collection, including clinical observation. SYMMACS data collection involved four instruments: (#1-a) a quality-assessment (QA) of the VMMC site (a shortened version of the WHO assessment tool [8]); (#1-b) observation of up to 10 VMMC procedures per site, (#2) provider interview; and (#3) compilation of monthly service statistics and presence of efficiency elements at the site, based on the site managers' recall (instruments available online [9]). Data were collected for all instuments during a two-day visit to each site in each year of data collection. Given this two-stage sampling, the number of cases in the tables and figures presented in this article differed by instrument. The unit of analysis was the site for the site assessment (instrument 1-a); the provider for the provider survey (instrument 2); and the VMMC procedures performed for the observation (instrument 1-b). In Figure 1, which shows the growth in number of sites in each country over time, the n per month depended on the number of sites that were operational in that month. (For example, if a mobile site was inactive in a given month, there would be nothing to report.)

Instruments 1-a and 1-b included checklists which clinician(s) on the team would score as “0” (unsatisfactory), “1” (partially satisfactory), or “2” (satisfactory), based on written guidelines and training exercises. The social scientist on the team generally conducted the provider interviews. Instrument #3 compiled data from existing service statistics starting from January 2010 (a full year prior to the onsite data collection for the other 3 instruments) to provide a longer trend line. The number of observations in 2012 differed by country, depending on last month of data collection; thus, the total observation period ranged from 29 months (RSA) to 33 months (ZW). In all countries, the Ministry of Health issued letters authorizing data collection, which took place between May and December in 2011 and between April and October in 2012 (with variations due to in-country logistical considerations).

For quality control, country coordinators reviewed clinicians' scoring following each day of data collection and met with their teams regularly to resolve problematic issues to ensure consistent assessment within teams. For instrument 3, the teams routinely verified data obtained from central offices with service statistics available at field sites. To ensure cross country consistency, the PI or assistant visited each country in both 2011 and 2012 to observe data collection, practice interviewing techniques, review terminiology, and discuss problems encountered by the teams.

Data from instruments 1-a, 1-b, and 2 were entered manually and on PDAs (model HP iPAQ 210) using the software Entryware Designer 6.4. Instrument 3 was entered manually into Excel and then Microsoft Access; queries were created in Access to produce the data needed for the graphs, and these data were exported to Excel for creation of the graphs.

### Data analysis

Data were analyzed in SPSS (v.19.0) and STATA (v.12); graphs were produced in Excel. Data in the figures showing monthly trends are descriptive. Using a one-way ANOVA we tested differences between countries in the number of surgical beds used in a given year and used the Bonferroni test of multiple comparisons to determine which countries were significantly different from others. An independent samples T-test was also used to assess differences within countries from 2011 to 2012. Task-sharing practices of providers across countries and years were compared using the Pearson's chi square and Fisher's exact test (expected cell frequencies were <5). Finally, 95% confidence intervals were calculated for all means and point estimates.

The original data analysis plan called for multivariate analysis across the four countries to explore relationships between the elements of efficiency and outcome variables such as total operating time or provider burnout. However, these efficiency elements were often implemented as a result of national policies and the country effect precluded analyses due to the lack of within-country variation and collinearrity between country and efficiency elements.

Ethics approval. The researchers obtained human subjects approval for this study from the Tulane University Institutional Review Board (IRB) and the local IRBs in each country; the Kenya Medical Research Institute, University of the Witwatersrand's Human Research Ethics Committee in South Africa, Tanzanian National Institute for Medical Research, and the Medical Research Council of Zimbabwe. All site managers and clinical service providers gave written consent to be interviewed, and in the case of the latter, to be observed performing VMMCs. Additional scientific and ethical reviews were done by the national AIDS coordinating bodies, other government officials, local NGOs, and other stakeholders. All those above-mentioned IRBs approved the full study.

## Results

Data were collected from 73 sites in 2011 and 122 sites in 2012 across the four countries (see Table 1). In KE, RSA, and TZ, the majority of sites were fixed facilities, in contrast to ZW where the majority were outreach. A total of 358 VMMC providers were interviewed in 2011, compared to 591 providers in 2012. SYMMACS observed 594 VMMC procedures in 2011 and 1034 in 2012. Given expansion of its program, RSA had a higher number of sites, providers interviews and VMMC procedures observed than the other countries.

**Table 1:** Number of sites visited, providers interviewed, and VMMC procedures observed, by year and by country.

		Kenya	South Africa	Tanzania	Zimbabwe	Total
# sites visited	2011	30	15	14	14	73
	2012	29	40	29	24	122
Breakdown of sites: fixed/outreach/mobile	2011	15/12/3	13/2/0	13/1/0	5/9/0	—
	2012	15/12/2	26/14/0	24/5/0	6/18/0	—
# provider interviews	2011	86	105	93	74	358
	2012	82	209	206	94	591
# of VMMC procedures observed	2011	151	120	133	140	594
	2012	218	361	251	204	1034

Figure 1 demonstrates different patterns in the scale-up of VMMC sites over the approximately 2.5 year period of observation. The number of sites shown were those sites that were sampled for the study and operational in that specific month. In KE, 30 sites were sampled, but data are shown only for months in which a given site was operational: at least 10 throughout 2010, increasing to over 25 throughout 2012. By contrast, in the other three countries, very few sites were operational as of January 2010: 1 (RSA), 2 (TZ), and 3 (ZW). The graphs for RSA and TZ reflect a steady increase in number of operational sites throughout 2011 and 2012, peaking at 31 and 27, respectively. In ZW, the number of fixed sites remained fairly constant over the observation period (between 2–5), but outreach sites increased markedly in 2011 and 2012, creating a peak of 23 total sites in April 2012.

**Figure 2:**
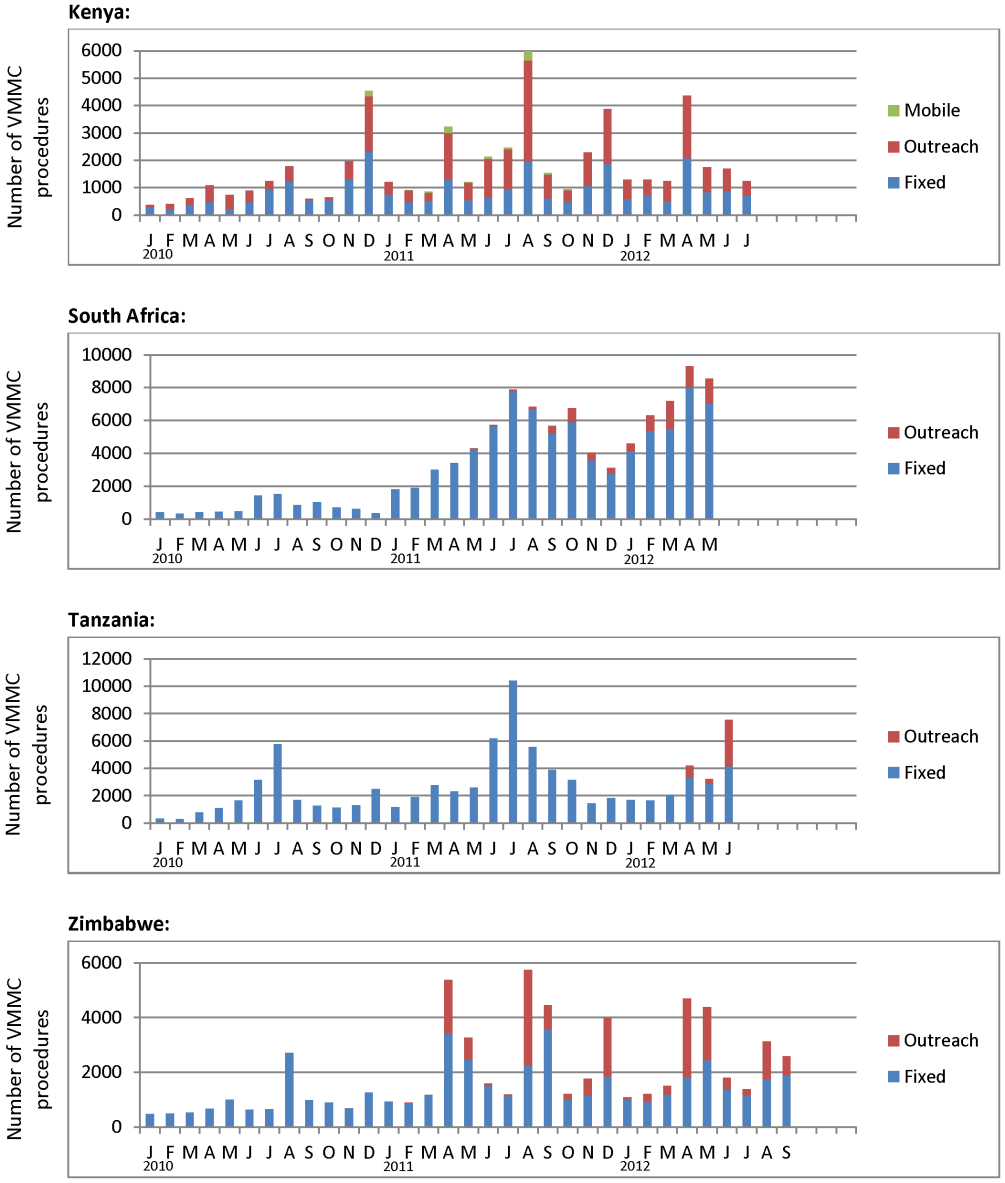
Number of VMMC procedures performed by month, type of site, and country, January 2010 – mid-2012.

**Figure 3:**
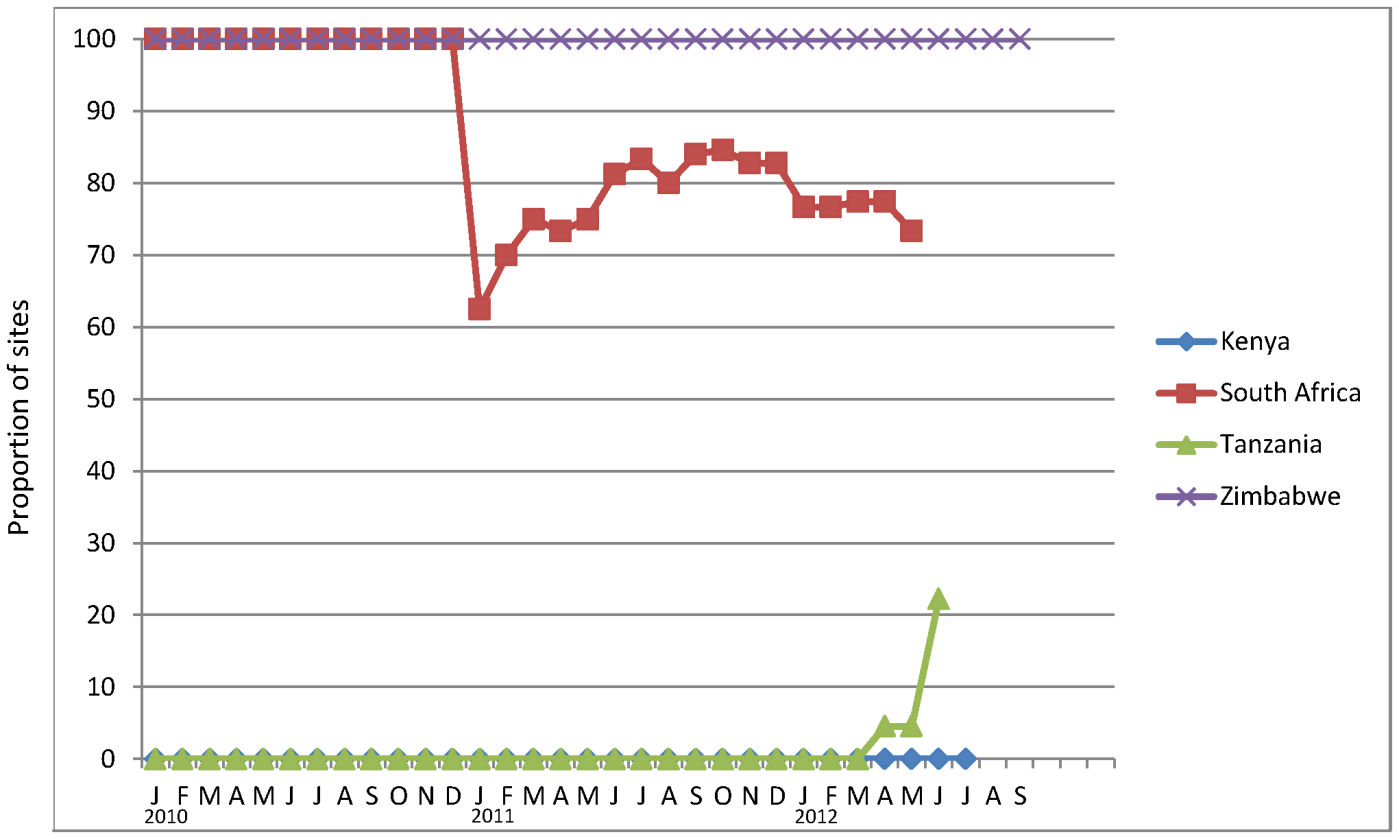
Proportion of VMMC sites using pre-packaged kits with consumables and disposable instruments, January 2010 – mid-2012. The number of sites on which these data are based varies markedly by month over the observation period, as shown in Figure 1.

**Figure 4:**
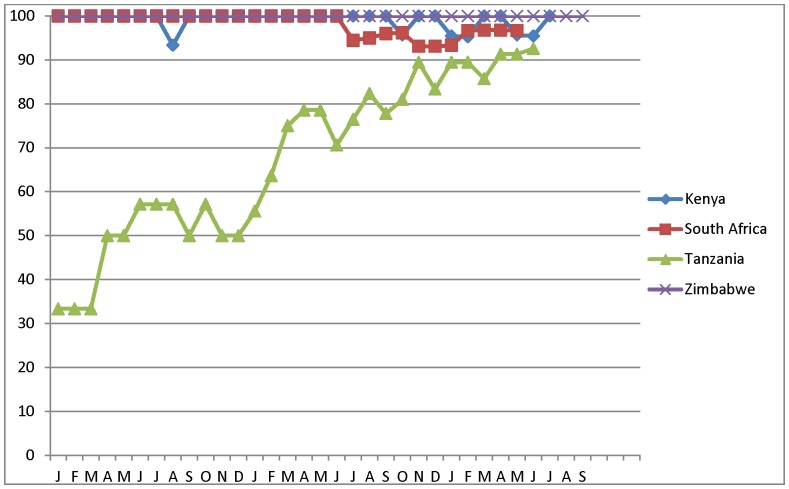
Proportion of VMMC sites by month and by country using forceps-guided method, January 2010 –mid-2012. The number of sites on which these data are based varies markedly by month over the observation period, as shown in Figure 1.

**Figure 5:**
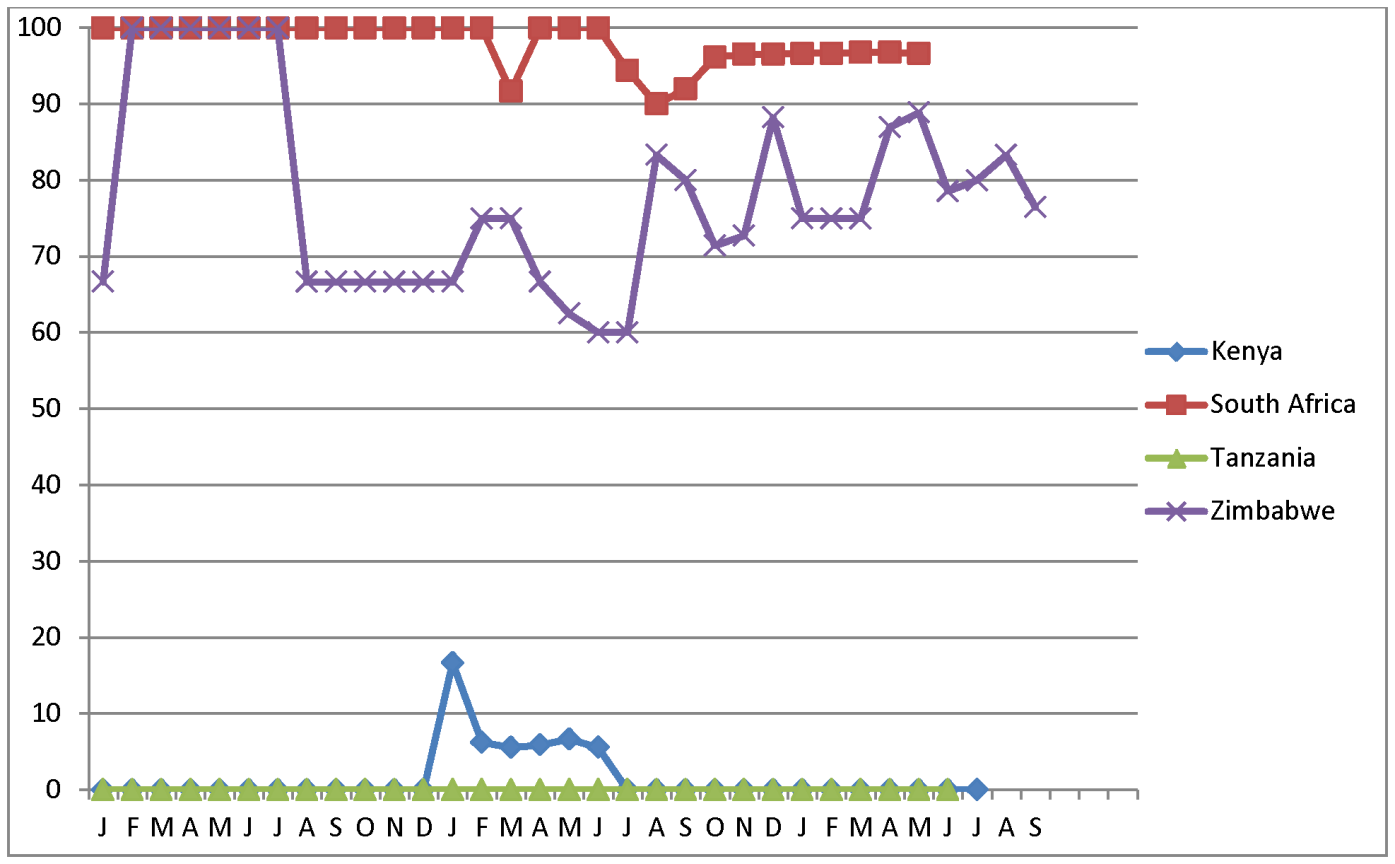
Proportion of sites by month and by country using electrocautery/diathermy, January 2010 – mid-2012. The number of sites on which these data are based varies markedly by month over the observation period, as shown in Figure 1.

**Figure 6:**
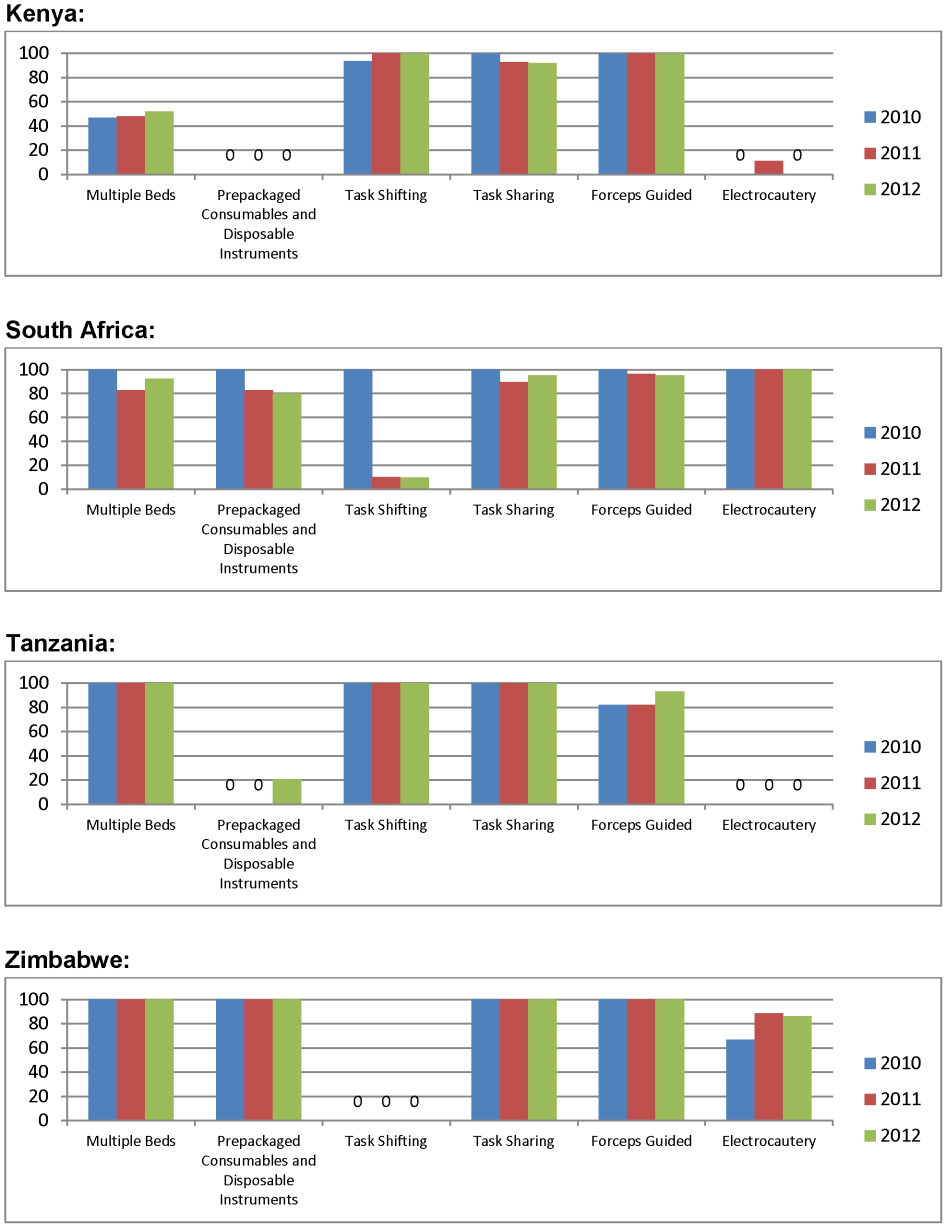
Proportion of sites adopting each efficiency element by year and by country.

All countries experienced dramatic seasonal fluctuations in monthly client load (Figure 2). Client load in KE peaked in July/August and November/December. RSA and TZ peaked in June/July; and ZW peaked in April/May, August/September, and December. Tables 2–3 and figures 3, 4, 5, and 6 describe the extent to which these four countries had adopted each of the six elements of surgical efficiency described above in 2011 and 2012.

**Table 2:** Surgical beds per site, by country and by year, based on site assessment.

Proportion of facilities with this number of surgical beds (bays):	Kenya		South Africa		Tanzania		Zimbabwe	
	2011	2012	2011	2012	2011	2012	2011	2012
	n = 30	n = 29	n = 15	n = 40	n = 14	n = 29	n = 14	n = 24
1	66.7	79.3	6.7	12.5	37.5	27.6	0.0	0.0
2	16.7	20.7	6.7	12.5	57.1	58.6	0.0	41.7
3	10.0	0.0	6.7	20.0	7.1	6.9	42.9	33.3
4	3.3	0.0	33.3	32.5	0.0	6.9	35.7	20.8
5	0.0	0.0	13.3	10.0	0.0	0.0	14.3	4.2
6	0.0	0.0	6.7	7.5	0.0	0.0	7.1	0.0
7	0.0	0.0	13.3	0.0	0.0	0.0	0.0	0.0
8	0.0	0.0	13.3	2.5	0.0	0.0	0.0	0.0
9	0.0	0.0	0.0	2.5	0.0	0.0	0.0	0.0
Mean number of beds per site*	1.4	1.2	4.8**	3.7**	1.7	1.9	3.9**	2.9**
	(1.1–1.7)^1^	(1.1–1.4)	(3.8–5.9)	(3.1–4.2)	(1.4–2.0)	(1.6–2.2)	(3.4–4.4)	(2.5–3.2)

% confidence intervals;^1^ 95

**p value (of differences between countries) <0.001; p value (of difference between years) <0.05.

**Table 3:** Task-sharing: percentage of providers reporting that secondary providers complete certain clinical tasks in the VMMC operating theater, by year and by country.

Tasks completed by secondary provider	Kenya		South Africa		Tanzania		Zimbabwe	
	2011	2012	2011	2012	2011	2012	2011	2012
	n = 85	n = 82	n = 105	n = 209	n = 93	n = 206	n = 74	n = 94
Administered local anesthesia	79.2*	58.5*	98.0	96.2	96.8	99.5	77.0**	98.9**
	(70.5–87.8)	(47.8–69.2)	(95.3–100)	(93.6–98.8)	(93.2–100)	(98.54–100)	(67.4–86.6)	(96.8–100)
Completed suturing of skin after primary provider removed the foreskin and achieved hemostasis	55.3	61.0	95.2	96.7	100.0	100.0	68.9**	90.4**
	(44.7–65.9)^1^	(50.4–71.6)	(91.1–99.3)	(94.3–99.1)	(100–100)	(100–100)	(58.4–79.45)	(84.4–96.4)

1 95% confidence intervals;

*p value (of difference between years) <0.05;

**p value (of difference between years) <0.001. Countries differed significantly (p<.001) on both variables.

### Multiple surgical bays

In both 2011 and 2012, the mean number of surgical beds in use for VMMC per site was higher in RSA (4.8, 3.7) and ZW (3.9, 2.9) than in KE (1.4, 1.2) and TZ (1.7, 1.9) (table 2; p<0.001). The decrease in mean number of beds per site between 2011 and 2012 in RSA and ZW was statistically significant (p<.05); it reflects the dramatic expansion of sites in RSA (with an increase in the smaller sites) [10] and increased use of outreach sites with fewer beds in ZW. The lower mean number of surgical beds in use in KE sites partially reflects low client load during data collection (which did not coincide with KE's Rapid Response Initiative). Data from the provider survey indicate that 100% of providers in ZW (2011 and 2012), 80–98% of providers in RSA and TZ (both years), and 60–64% among providers in KE (both years) reported rotating among surgical beds.

### Pre-packaged kits with consumables and disposable instruments

Figure 3 shows the proportion of sites by month and by country with at least 80% of procedures conducted using pre-packaged kits with disposable instruments. ZW used such kits from the start of its program at all sites. RSA used kits in its two sites operational in 2010; the sharp drop in January 2011 reflects the inclusion of new sites, some of which did not use disposable instruments or used them in addition to reusable instruments. In TZ, VMMC kits with disposable instruments were introduced into SYMMACS sites only at the end of 2012. And KE reused its instruments throughout the observation period.

### Task-shifting (allowing trained other clinical providers to conduct the entire VMMC procedure)

Countries took an “all or none” approach to task-shifting, based on national policy: “all” in KE and TZ but “none” in ZW. In RSA, task-shifting was not authorized but occurred sporadically at the 1–2 clinics that operated in 2010 but to a lesser extent as more clinics became operational in 2011-12 (Figure 5). The ratio of medical doctors to other clinical providers averaged 1∶3.5 and 1∶4.7 in RSA, and 1∶2.7 and 1∶2.8 in ZW in 2011 and 2012, respectively.

### Task-sharing (other clinical providers authorized to conduct certain aspects of the procedure)

Task-sharing was nearly universal in all countries by 2012, except in KE. Based on the provider interview, by 2012 over 96% of providers in all countries reported that secondary providers administered local anesthesia; Kenya was lower (79% in 2011, 59% in 2012), because of a preference for having a single provider operate on one client from start to finish. The percentage of providers that reported that secondary providers completed suturing after the primary provider had removed the foreskin and achieved hemostatis was at least 90% by 2012 in all countries except KE (where it was 55% in 2011, 61% in 2012); see Table 3.

### Surgical method

The vast majority of providers – over 96% in all countries and both years – reported to use forceps-guided in at least 90% of procedures. Site managers in TZ reported some use of dorsal slit in the few sites operating in 2010, based on training received in another country, but use of forceps-guided increased steadily starting in 2011 (Figure 4).

### Use of electrocautery (diathermy) instead of ligating sutures

Figure 5 reflects the differing extent of adoption of electrocautery by country, based on the monthly data. Electrocautery was used in almost all sites in RSA, the majorityof sites in ZW, very few sites in KE, and at no sites among those sampled in TZ. Results from the provider survey confirmed these observations. Over 98% of providers in RSA (both years) used electrocautery for hemostasis in performing or assisting in performing VMMC. In ZW, the percentage increased from 72% to 92% between 2011 and 2012. In sharp contrast, only 33% of providers in KE had ever used it in 2011, dropping to 20% in 2012. And in TZ, no providers in either year reported having used this technique.

### Overview of the extent of adoption of the six efficiency elements across countries

Figure 6 – summarizing the extent of adoption of the six surgical efficiency elements – shows that none of the four countries had adopted all six elements. RSA had adopted five and ZW was moving toward adoption of five. KE and TZ– pioneers in VMMC task-shifting – had not adopted either purchased kits with disposable instruments or electrocautery; and KE showed minimal use of multiple surgical beds. In short, major differences occurred by country, with relative uniformity within country, reflective of national policies. The few cases og within-country variation included TZ (decrease in the use of dorsal slit in 2010 toward forceps-guided, and increase in use of pre-bundled kits with disposable instruments in 2012) and in RSA (occasional task-shifting in a country where it is not authorized). The only element adopted across all four countries was use of forceps-guided as the surgical method.

## Discussion

When WHO described efficiency models for VMMC programs in 2010, countries had little empirical evidence on the performance of these elements [7]. SYMMACS addressed this evidence gap by providing data on the use of the six surgical efficiency elements in field settings in four highly active VMMC programs. The findings have provided useful information to host governments, implementing agencies, donors, and health services researchers. Of note, no country had adopted all six elements, although RSA and ZW came closest. However, neither had adopted task-shifting, which arguably is a critical element in meeting ambitious country targets for VMMC.

The study had a number of limitations. The sampling was designed to capture VMMC sites as they became operational to maximize the number included; it was not possible to establish the universe of sites and randomly select from them (except in Kenya). In the other three countries, additional sites were added using purposeful sampling, based on factors that were priorities for each country but not standard across countries Lack of information about services offered at government sites and the pace of expansion made sampling particularly difficult in RSA. Ideally, data collection would have occurred in high volume periods when efficiency elements would be most beneficial; however, data collection was constrained by a number of logistical factors specific to each country. As a result, the data collection occurred under varied conditions – a mix of high, moderate and low volume client loads – except in ZW in 2011 (high) and RSA in 2012 (low). The observations of sites and VMMC procedures were by definition subjective, although based on pre-established written criteria. Because of the multi-country data collection and training of data collecters in-country, it was not possible to test inter-rater reliability in the scoring of quality for the site assessments and for observation of VMMC procedures. Moreover, in some cases clinicians who had trained VMMC providers at an earlier data formed part of the SYMMACS data collection teams, which could have introduced bias. However, in one country where such an individual (who was not only a physician but a surgeon) was part of the data collection team, the bias may have been in the direction of being more severe in his assessment of the facilities and VMMC procedures performed.

SYMMACS results contribute to the scant literature on VMMC service delivery: on human resource issues [11], [12], disposable versus reusable instruments [13], the safety of task-shifting [14], and descriptions of programs implementing efficiency elements in KE [15], RSA [16], TZ [17], and Uganda [18]. In addition, they have prompted policy debate and programmatic action in all four countries where they have been presented in in-country diseemination conferences, scientific meetings, workshops, and other venues.

The SYMMACS results have produced some actions that are common across all countries as well as others that are specific to each country. In all four countries, program implementers and policy-makers have developed a keen awarenes of the need to enhance mechanisms to monitor the quality and safety of their rapidly expanding VMMC programs. SYMMACS identified strengths as well areas for improvement, which provided concrete action items for training, supervision, information systems, and other areas that contribute to quality and safety in service delivery. SYMMACS provided the benefits of a quality monitoring system where no external form of systemactic monitoring previously existed.

In terms of country-specific results, in KE the results have contributed to revamping of the theatre environment, use of commercially-bundled kits with disposable instruments, and more frequent supervisory visits to assure quality. Discussions are underway to include items for tracking the quality of VMMC services into the MOH tool for routine supervision of healthcare services. In TZ, the results also facilitied a shift towards increased use of commercially bundled VMMC kits. Stakeholders in TZ found it useful to know where they stood as a country vis-à-vis other countries and international standards; the results served as an additional motivation for moving the program forward.

In RSA, as reported elsewhere in this supplement [10], SYMMACS data prompted local authorities to re-examine the national system to track adverse events, as well as introduce a systematic and standardised monitoring and evaluation system. Furthermore, two workshops held with stakeholders and government officials involved in the RSA VMMC programmefocused on ways to improve program shortcomings, specifically, the lack of emergency equipment at many of the sites, the limited access to appropriate guidelines, the unsatisfactory systsems for monitoring of adverse events, and the absence of appropriate external supervision at many of the sites. Stakeholders and government officials are currently putting in corrective measures in their programmes these areas found lacking.

One of the most notable changes to which SYMMACS contributed was in ZW, which in late late 2012 reversed its policy against task-shifting and is currently piloting a project that will allow trained nurses to perform VMMC (as is currently done in KE and TZ). Task-shifting will enhance the country capacity to expand its VMMC programming to all 62 health districts in the country. Preliminary findings – yet to be published - suggest that nurses who have undergone careful training (and in most cases had previously assisted in performing VMMC) are able to provide VMMC at satisfactory levels of quality and safety (Hatzold, personal communication).
